# Changes in gene expression of histone modification enzymes in rat granulosa cells undergoing luteinization during ovulation

**DOI:** 10.1186/s13048-016-0225-z

**Published:** 2016-03-15

**Authors:** Ryo Maekawa, Lifa Lee, Maki Okada, Hiromi Asada, Masahiro Shinagawa, Isao Tamura, Shun Sato, Hiroshi Tamura, Norihiro Sugino

**Affiliations:** Department of Obstetrics and Gynecology, Yamaguchi University Graduate School of Medicine, Minamikogushi 1-1-1, Ube, 755-8505 Japan

**Keywords:** Histone modification, Granulosa cells, Ovulation, Luteinization

## Abstract

**Background:**

The ovulatory LH surge rapidly alters the expression of steroidogenesis-related genes such as steroidogenic acute regulatory protein (*StAR*) in granulosa cells (GCs) undergoing luteinization. We recently reported that histone modifications contribute to these changes. Histone modifications are regulated by a variety of histone modification enzymes. This study investigated the changes in gene expression of histone modification enzymes in rat GCs undergoing luteinization after the induction of ovulation. The extracellular regulated kinase (ERK)-1/2 is a mediator in the intracellular signaling pathway stimulated by the ovulatory LH surge and regulates the expression of a number of genes in GCs. We further investigated whether ERK-1/2 is involved in the regulation of the histone modification at the *StAR* promoter region in GCs undergoing luteinization.

**Results:**

GCs were obtained from rats treated with equine chorionic gonadotropin (CG) before (0 h) and after human (h) CG injection. The expressions of 84 genes regulating histone modifications or DNA methylation were measured using a PCR array. Five genes (*HDAC4, HDAC10, EZH2, SETDB2, and CIITA*) were identified as histone acetylation- or histone methylation-related genes, and were significantly altered after hCG injection. None of the genes were related to DNA methylation. mRNA levels of *EZH2, SETDB2, HDAC4*, and *HDAC10* decreased and *CIITA* mRNA levels increased 4 or 12 h after hCG injection.

GCs isolated after eCG injection were incubated with hCG for 4 h to induce luteinization. *StAR* mRNA levels were significantly increased by hCG accompanied by the increase in H3K4me3 of the *StAR* promoter region. *StAR* mRNA expression was inhibited by the ERK inhibitor with the significant decrease of H3K4me3. These results suggest that hCG increases *StAR* gene expression through the ERK-1/2-mediated signaling which is also associated with histone modification of the promoter region.

**Conclusions:**

Gene expressions of histone modification enzymes change in GCs undergoing luteinization after ovulation induction. This change may play important roles in regulating the expression of various genes during the early stage of luteinization, which may be critical for the subsequent corpus luteum formation.

## Background

The ovulatory LH surge alters the expression of various genes, which are essential for ovulation, in granulosa cells undergoing luteinization [1]. Among the genes involved in steroidogenesis, the expression of steroidogenic acute regulatory (*StAR*) protein, a rate-limiting enzyme for progesterone synthesis, is rapidly induced after the LH surge [2] while the expression of aromatase (*Cyp19a1*), a key enzyme for estrogen synthesis, is rapidly suppressed [3]. This functional change of steroidogenesis, in which there is a rapid shift from estrogen synthesis to progesterone synthesis, plays a crucial role in follicle rupture and the following corpus luteum formation [4, 5].

In recent years, epigenetic mechanisms such as DNA methylation and histone modifications have been shown to be involved in the regulation of gene expression [6–9]. DNA methylation occurs at cytosines within CpG dinucleotides through DNA methyltransferases (DNMT), which can silence gene expression by altering chromatin structure and preventing the binding of transcription factors [10–13]. Histone modifications also affect chromatin structure, which is critical for the interaction of transcriptional factors with response elements in the promoters [10, 14–16]. Histone modifications such as acetylation of histone H3 and histone H4 or trimethylation of the site of lysine 4 on histone H3 (H3K4me3) activate transcription by loosening the chromatin structure and allowing the recruitment of transcriptional factors to their response elements [17–19]. On the other hand, histone modifications such as trimethylation of the site of lysines 9 and 27 on histone H3 (H3K9me and H3K27me3) inactivate transcription by condensing the chromatin [20, 21].

Histone modifications have been reported to regulate the gene expression of *StAR*, steroidogenesis-related enzymes, and LH receptor in the ovary [22–24]. We recently reported that histone modifications and chromatin remodeling contribute to the rapid induction of the *StAR* gene and the rapid suppression of the *Cyp19a1* gene in granulosa cells undergoing luteinization during ovulation in rats [25]. Levels of histone H4 acetylation and H3K4me3 increased whereas H3K9me3 and H3K27me3 decreased in the *StAR* promoter after ovulation induction [25]. On the other hand, the levels of histone H3/H4 acetylation and H3K4me3 decreased, and the level of H3K27me3 increased in the *cyp19a1* promoter after ovulation induction [25]. Histone modifications are regulated by a variety of histone modification enzymes. Recently, CREB-binding protein (CBP)/p300 was shown to regulate the histone acetylation in the promoters of the LH-induced target genes as a histone acetyltransferase in mouse granulosa cells undergoing luteinization after the ovulatory LH surge [26]. However, little information is available on how and which histone modification enzymes change in granulosa cells undergoing luteinization.

The extracellular regulated kinase (ERK)-1/2 is a mediator in the intracellular signaling pathway stimulated by the ovulatory LH surge and regulates the expression of a number of genes including *StAR* and *cyp19a1* in granulosa cells [27]. Recent studies have shown that LH surge-related signaling pathways regulate the expression of some histone modification enzymes. CBP/p300, a histone acetyltransferase, acts downstream of the ERK-1/2 signal activated by the ovulatory LH surge in mouse granulosa cells [26]. Two genes (*NCOA7* and *HP1BP3*), which are related to histone modifications or chromatin remodeling, also act downstream of the ERK-1/2 signal and are involved in luteinization of granulosa cells after ovulation induction [27]. These findings led us to investigate that ERK-1/2 is involved in regulating the gene expression through histone modifications in granulosa cells undergoing luteinization after the ovulatory LH surge.

In this study, we first investigated the changes in gene expression of histone modification enzymes in rat granulosa cells undergoing luteinization after ovulation induction. Furthermore, we investigated whether ERK-1/2 is involved in the regulation of the histone modification at the *StAR* promoter region and its mRNA expression in granulosa cells undergoing luteinization.

## Methods

The present study was reviewed and approved by the Committee for the Ethics on Animal Experiment in Yamaguchi University Graduate School of Medicine.

### Animal models

Female Sprague–Dawley rats (Japan SLC, Hamamatsu, Japan) in 21–24 days old were injected subcutaneously with 15 IU of equine chorionic gonadotropin (eCG) (Sigma, St. Louis, MO, USA) to stimulate follicular growth. After 48 h, the rats were injected subcutaneously with 15 IU of human chorionic gonadotropin (hCG) (Sigma) to induce ovulation and luteinization. The ovaries were dissected before hCG (0 h), 4 and 12 h after hCG injection. Then, follicles were punctured using 27 G needles, and granulosa cells were isolated. The cells were centrifuged at 800 x g, pelleted, and washed in cold PBS twice. Total RNA was isolated from the cells with Isogen (Wako Pure Chemical Industries Ltd., Osaka) and purified by RNeasy Mini (QIAGEN, Chatsworth, CA). Three to five rats in each time point were used for RT2 profiler PCR array or real-time reverse transcription (RT)-PCR described below.

### Collection and culture of granulosa cells

Culture of granulosa cells was performed as reported previously [28]. Immature (3 weeks) ICR mice (Japan SLC Inc.) received a subcutaneous injection of 20 units of eCG to stimulate the development of multiple follicles. Mice were laparotomized under deep ether anesthesia 48 h after the eCG injection; the ovaries were quickly removed for the following experiments, and the mice were euthanized by exsanguinations. The ovaries were transferred to alpha Modified Eagle Minimum Essential Medium supplemented with penicillin-streptomycin (Invitrogen, Carlsbad, CA, USA). Granulosa cells were collected by puncturing mature preovulatory follicles using a 27 G needle under a dissecting microscope. The cells were centrifuged at 800 x g, washed in PBS twice, and used for cell culture. The cells (at a density of 2.5 × 10^4^ cells/well in 100 μl of medium) were incubated with 50 units of hCG for 4 h to induce luteinization. In this study, high dose of hCG was used to significantly stimulate *StAR* gene expression. In addition, to investigate the involvement of the ERK-1/2 signal in the *StAR* expression, cells were incubated with the inhibitor of ERK-1/2 (U0126, 10 μM; Cell Signaling Technologies, Danvers, MA, USA) for 2 h prior to hCG stimulation. After incubation, the cells were used for evaluation of *StAR* mRNA expression and H3K4me3 status of the *StAR* promoter region.

### Real-time PCR array

RT of RNA into cDNA was performed using the RT^2^ First Strand Kit (QIAGEN) in accordance with the instruction manual. In this study, the RT^2^*Profiler*™ PCR Array, ‘Human Epigenetic Chromatin Modification Enzymes’ (QIAGEN) covering 84 genes that are related to histone modifications and DNA methylation, was used as we previously reported [29]. Real-time PCR was performed using the RT^2^ SYBR Green Master Mix (QIAGEN) according to the manufacturer’s protocol under the following cycler conditions: 95 °C: 10 min; 40 cycles (95 °C: 15 s; 60 °C: 60s) using the Applied Biosystems 7700 Real-time PCR cycler (Applied Biosystems, Darmstadt, Germany). The relative quantity of cDNA was calculated with the ΔΔC_t_ method using five normalization genes: β-2-microglobulin, hypoxanthine phosphoribosyltransferase 1, ribosomal protein L13a, glyceraldehydes-3-phosphate dehydrogenase and β actin. A significant change in gene expression among the times examined was defined as at least a 2-fold up- or down-regulation of genes with *p* < 0.01.

### Real-time RT-PCR

Total RNA was isolated from cells with Isogen (Wako Pure Chemical Industries Ltd., Osaka, Japan), and real-time RT-PCR was performed as we reported previously [25], using LightCycler with Premix-Ex-Taq (Takara, Ohtsu, Japan) and sequence-specific primer sets of enhancer of zeste homolog 2 (EZH2), SET domain-bifurcated 2 (SETDB2), histone deacetylase 4 (HDAC4), HDAC10, class II, major histocompatibility complex, transactivator (CIITA), nuclear receptor coactivator 7 (NCOA7), heterochromatin protein 1-binding protein 3 (HP1BP3), StAR and GAPDH (Table 1). The thermocycling program was 40 cycles of 95 C for 5 s and 60 C for 20 s with an initial cycle of 95 C for 10 s. RT-PCR amplification was also performed using a programmed temperature control system (PC808, ASTEC, Fukuoka, Japan). PCR products were separated on 2 % agarose-gel.

**Table 1:** Primers used for real-time RT PCR

Gene		Primer (5' to 3')	Amplification size (bp)
EZH2	Forward	GCTCTTTTGTCGACGATGTTT	63
	Reverse	TTGGGTGTTGCATGAAAGG	
SETDB2	Forward	GCAACACCAAAAGATGGAAGA	65
	Reverse	CATCTTGTAGCTCCATCCAGAA	
HDAC10	Forward	CGATGTGTAGCCCATAGAGGT	93
	Reverse	CCACAGAATTCTCCCATTGC	
HDAC4	Forward	CACACCTCTTGGAGGGTACAA	68
	Reverse	AGCCCATCAGCTGTTTTGTC	
CIITA	Forward	TCCTTCCAGCATTCTCTTCC	65
	Reverse	CCCGATCTTGTTCTCGCTAA	
NCOA7	Forward	CCACCAAGAGCTGGGAGAT	76
	Reverse	TCCTCCTCATAGTAGCTGCAAGT	
HP1BP3	Forward	TGAAGGGGAAGAAGAAAAACC	93
	Reverse	GGAGCAGGTGGAGTCTCATT	
STAR	Forward	GAAAGCCAGCAGGAGAATGG	78
	Reverse	CACCTCCAGTCGGAACACCTT	
GAPDH	Forward	CTCATGACCACAGTCCATGC	155
	Reverse	TTCAGCTCTGGGATGACCTT	

### Chromatin immunoprecipitation assay (ChIP assay)

ChIP assays were performed according to the protocol for the ChIP assay kit (Upstate Biotechnology, Lake Placid, NY, USA) as reported previously [25] with some modifications. Cells were cross-linked by addition of formaldehyde into the medium at a final concentration of 1 % and incubated for 10 min at 37 °C. Cross-linking was terminated by addition of glycine (0.125 M, final concentration). Cells were washed with ice-cold PBS containing protease inhibitors (Sigma) and resuspended in ChIP lysis buffer (1 % SDS, 10 mM EDTA, 50 mM Tris–HCl, pH 8.0, with protease inhibitors). The lysates were sonicated using a Bioruptor ultrasonicator (Cosmo-bio, Tokyo, Japan). After preclearing the lysate with salmon sperm DNA-protein A at 4 °C for 4 h, lysates were diluted with ChIP dilution buffer (0.01 % SDS, 1.1 % Triton X-100, 1.2 mM EDTA, 16.7 mM Tris–HCl, pH 8.0, 167 mM NaCl, with protease inhibitors) and 5 % of the supernatant were kept as input controls (INPUT). Dynabeads Protein A (Invitrogen) were incubated with antibodies for EZH2 (Santa Cruz Biotechnology, Inc., Santa Cruz, CA), H3K4me3 (generous gift from Dr. Kimura, Osaka University, Osaka, Japan) and normal rabbit IgG (Invitrogen) 4 °C overnight. The precleared chromatin was incubated with antibody-bound Dynabeads for 6 h at 4 °C. Immune complexes were collected and washed once for 5 min on a rotating platform with 1 ml each of the following buffers in sequence: low salt wash buffer (0.1 % SDS, 1 % Triton X-100, 2 mM EDTA, 20 mM Tris–HCl, pH 8.0, 150 mM NaCl), high salt wash buffer (0.1 % SDS, 1 % Triton X-100, 2 mM EDTA, 20 mM Tris–HCl, pH 8.0, 1500 mM NaCl), LiCl wash buffer (250 mM LiCl, 1 % Nonidet P-40, 1 % sodium deoxycholate, 1 mM EDTA, 10 mM Tris–HCl, pH 8.0), and twice with TE (10 mM Tris–HCl, pH 8.0, 1 mM EDTA). Immune complexes were eluted with 200 μl elution buffer (1 % SDS, 0.1 M NaHCO_3_, 10 mM DTT). Cross-linking of the immunoprecipitated chromatin complexes (IP) and INPUT were reversed by heating the samples at 65 °C overnight and subjected to proteinase K treatment. The DNA fragments were purified using a QIAquick PCR purification kit (QIAGEN) and subjected as a template for PCR amplification. The specific primers were used for ChIP-PCR analysis to amplify the *StAR* promoter region as reported previously [25]. To determine the relative levels of EZH2 recruitment and H3K4me3 statuses of the *StAR* promoter region, real-time PCR analysis was performed and the ratio of IP DNA to the INPUT DNA sample (%INPUT) was calculated as reported previously [25].

### Statistical analysis

In the real-time PCR array, a paired *t*-test (0 h vs 4 h and 0 h vs 12 h) was used to identify statistical differences for each transcript. All statistical analyses were performed using a web-based PCR array data analysis which is provided by the manufacture (Qiagen). Differences were considered significant at *p* < 0.01. In the RT-PCR, statistical significance was determined by one-way ANOVA. After ANOVA, the Tukey-Kramer test was applied to analyze differences between groups. In the in vitro study, unpaired *t* test was applied to analyze differences between two groups. All statistical analyses were performed using SPSS for Windows version 11 (SPSS Inc., Chicago, IL). Differences were considered significant at *p* < 0.05.

## Results

Among the 84 genes in the PCR array, three genes were up- or down-regulated by a factor of at least 2-fold between 0 and 4 h; *RPS6KA5* was up-regulated and *HDAC10* and *SETDB2* were down-regulated (Table 2). In addition, two genes (*NEK6* and *CIITA*) were up-regulated and eight genes (*AURKC, AURKB, HDAC10, SETDB2, HDAC4, EZH2, PAK1* and *CDK2*) were down-regulated by at least 2-fold between 0 and 12 h; (Table 3). All of these genes were related to histone modifications, but none of them were related to DNA methylation such as *DNMT1, DNMT3a,* and *DNMT3b*.

**Table 2:** Genes with a 2-fold up- or down-regulation between 0 and 4 h

Genes	Modifications	Fold	*P* value
Upregulated			
*RPS6KA5 (Ribosomal protein S6 kinase, aipha5)*	Histone Phosphorylation	3.74	0.0042
Downregulated			
*HDAC10 (Histone deacetylase 10)*	Histone Deacetylases	−2.17	0.0054
*SETDB2 (SET domain, bifurcated 2)*	Histone Methyltransferases	−2.08	0.0075

**Table 3:** Genes with a 2-fold up- or down-regulation between 0 and 12 h

Genes	Modifications	Fold	*P* value
Upregulated			
*NEK6 (NIMA (never in mitosis gene a)-related kinase 6)*	Histone Phosphorylation	2.02	0.0002
*CIITA (Class II, major histocompatibility complex, transactivator)*	Histone Acetyltransferases	2.29	0.0085
Downregulated			
*AURKC (Aurora kinase C)*	Histone Phosphorylation	−3.51	0.0008
*AURKB (Aurora kinase B)*	Histone Phosphorylation	−2.83	0.0017
*HDAC10 (Histone deacetylase 10)*	Histone Deacetylases	−2.65	0.0026
*SETDB2 (SET domain, bifurcated 2)*	Histone Methyltransferases	−3.11	0.0030
*HDAC4 (Histone deacetylase 4)*	Histone Deacetylases	−2.14	0.0029
*EZH2 (Enhancer of zeste homolog 2 (Drosophila))*	Histone Methyltransferases:	−2.55	0.0050
*PAK1 (P21 protein (Cdc42/Rac)-activated kinase 1)*	Histone Phosphorylation	−4.21	0.0044
*CDK2 (Cyclin dependent kinase 2)*	Histone Phosphorylation	−2.00	0.0099

**Fig. 1:**
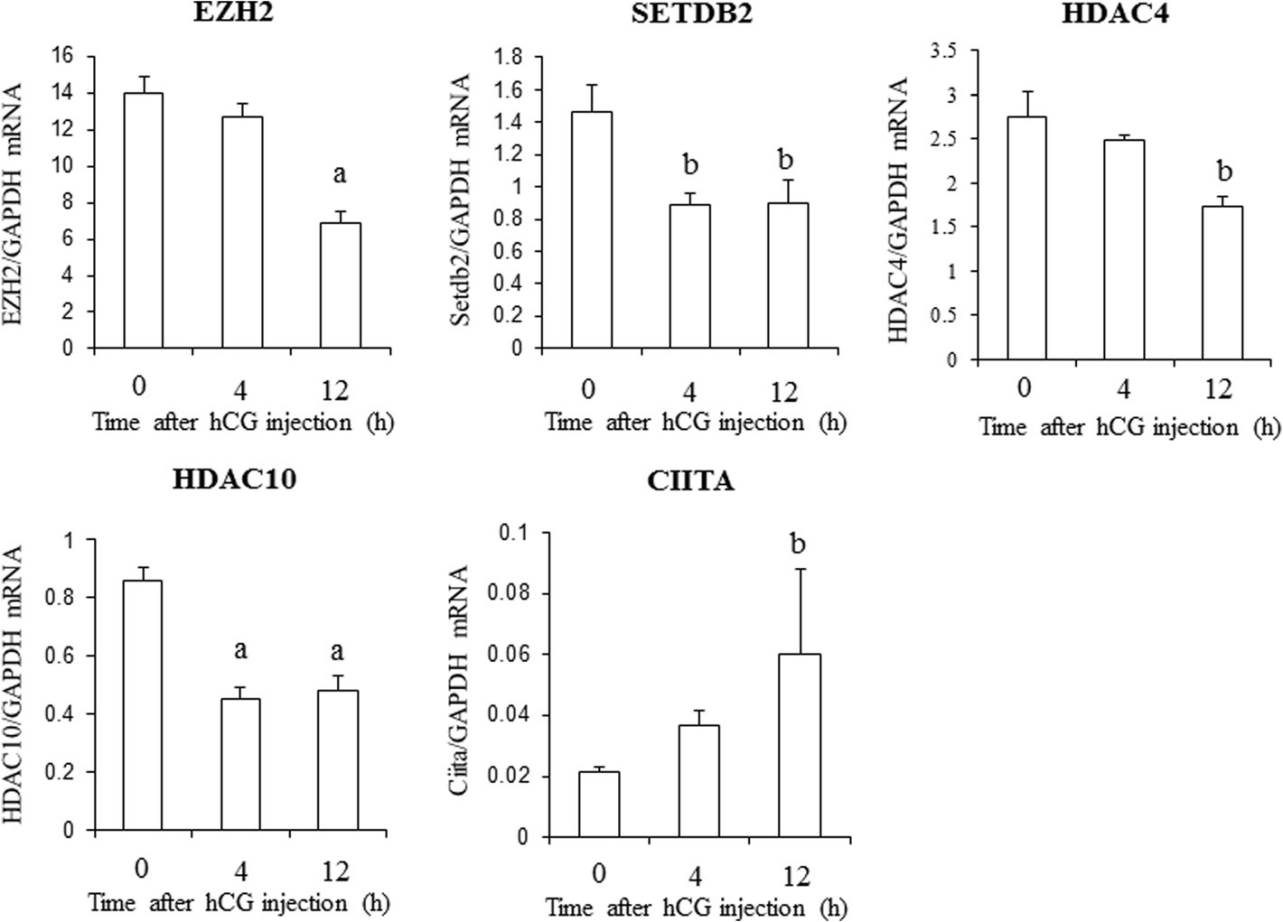
mRNA expression of *EZH2, SETDB2, HDAC4, HDAC10*, and *CIITA* in rat granulosa cells undergoing luteinization after ovulation induction. Granulosa cells were obtained from rats treated with eCG before (0 h), 4, and 12 h after hCG injection. *EZH2, SETDB2, HDAC4, HDAC10*, and *CIITA* were identified as genes related to histone acetylation or histone methylation, and validated with additional samples (*n* = 5 in each time point) by real-time RT-PCR. The value of mRNA of each gene was normalized to that of an internal control (*GADPH*). Data were expressed as a ratio of mRNA of each gene to *GADPH*. Each bar represents the mean +/− SEM of five animals. a; *p* < 0.01 vs. 0 h, b; *p* < 0.05 vs. 0 h

Five (*EZH2, SETDB2, HDAC4, HDAC10,* and *CIITA*) of the up- or down-regulated genes were related to histone acetylation or histone methylation. The changes in mRNA levels of the five genes were validated with additional samples (*n* = 5 in each time point) by real-time RT-PCR. EZH2 forms the polycomb-repressive complex 2 with other components and serves as the enzyme that induces H3K27me3 leading to gene silencing [30, 31]. The mRNA expression level of *EZH2* significantly decreased between 0 and 12 h (Fig. 1). SETDB2 has transcriptional repression activities through inducing H3K9me3 [32]. The mRNA expression of *SETDB2* significantly decreased at 4 and 12 h after hCG injection (Fig. 1). HDACs remove acetyl groups from acetylated histones and lead to inactivation of gene expressions [33]. HDAC4 and HDAC10 belong to class II histone deacetylases and have tissue-specific expressions [34]. *HDAC4* is most strongly expressed in heart, skeletal muscle and brain while *HDAC10* is expressed in liver, spleen and kidney [34]. The mRNA levels of *HDAC4* and *HDAC10* significantly decreased at 12 h, and at 4 and 12 h after hCG injection, respectively (Fig. 1). CIITA is a master regulator of major histocompatibility complex (MHC) class II genes and works as a histone acetyltransferase [35]. The mRNA expression of *CIITA* significantly increased at 12 h (Fig. 1).

**Fig. 2:**
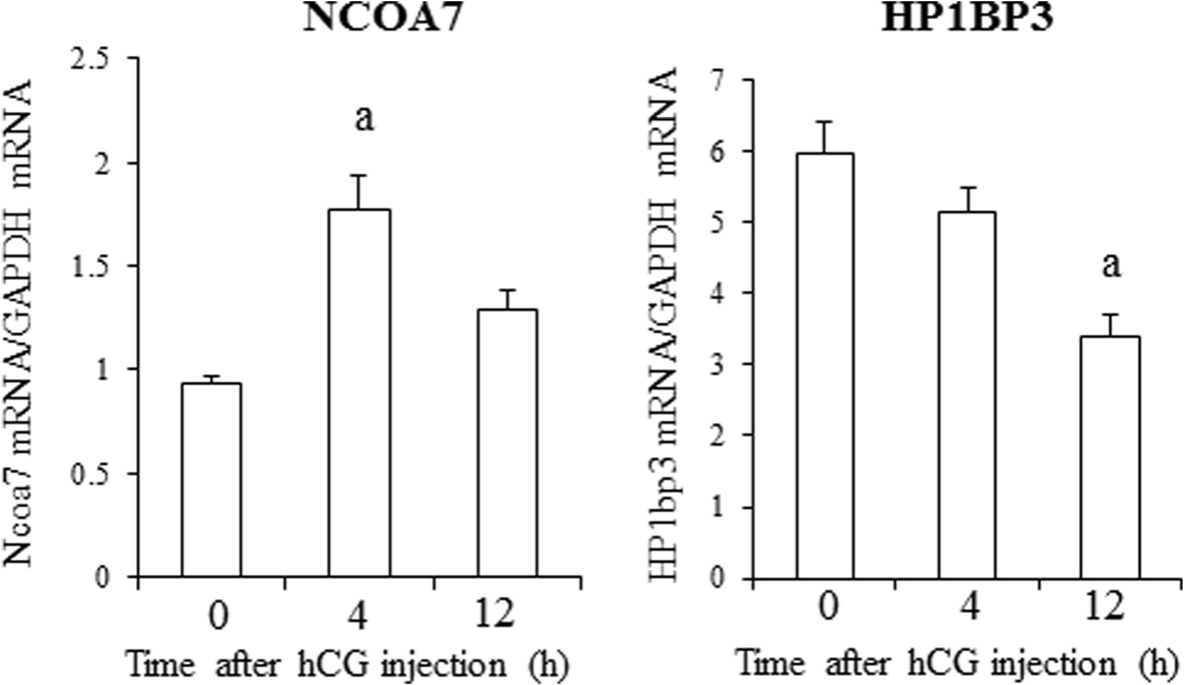
mRNA expression of *NCOA7* and *HP1BP3* in granulosa cells undergoing luteinization after ovulation induction. *NCOA7* and *HP1BP3* are related to histone modification or chromatin remodeling and act downstream of the ERK1/2 signal after ovulation induction. mRNA expressions of both genes were analyzed in the same model as described in Figure 1. The value of mRNA of each gene was normalized to that of the internal control (*GADPH*). Each bar represents the mean +/− SEM of five animals. a; *p* < 0.01 vs. 0 h

Two genes (*NCOA7* and *HP1BP3*) are related to histone modifications or chromatin remodeling, and are involved in luteinization of granulosa cells after ovulation induction by working downstream of the ERK-1/2 signal [27]. NCOA7 is an estrogen receptor coactivator that possesses intrinsic histone acetyltransferase activities [36, 37]. HP1BP3 is a component of heterochromatin that maintains heterochromatin integrity [38, 39]. Since these two genes were not included in the PCR array used in this study, we examined them separately. The mRNA expression level of *NCOA7* significantly increased 4 h after hCG injection, while the mRNA expression level of *HP1BP3* significantly decreased 12 h after hCG injection (Fig. 2).

**Fig. 3:**
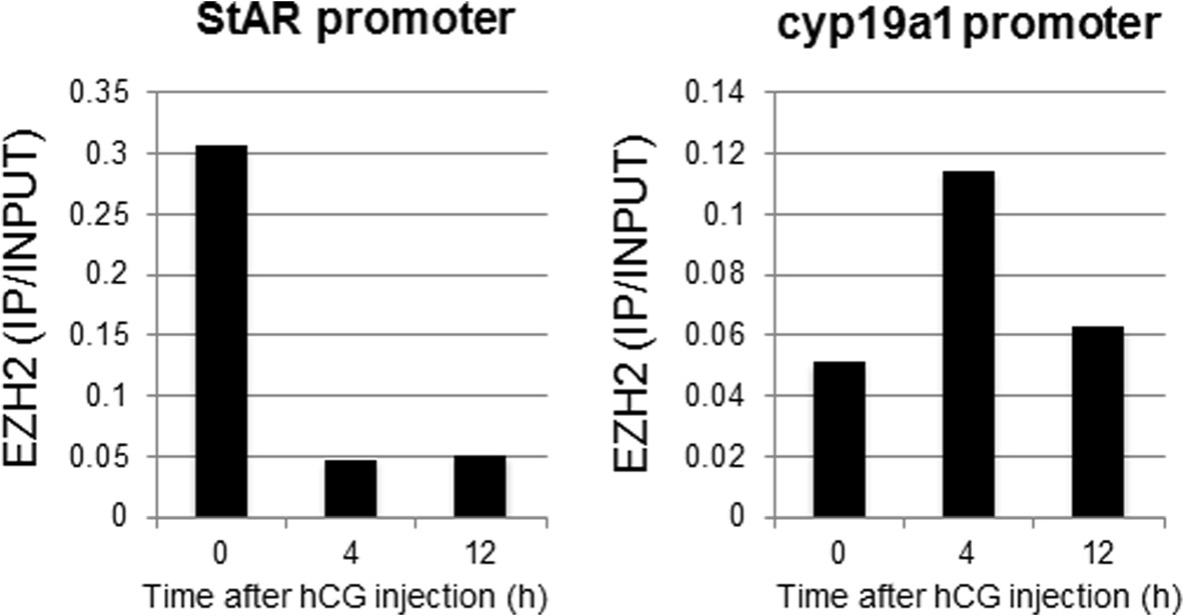
Recruitment of EZH2 to the promoter regions of *StAR* and *cyp19a1*. Granulosa cells were obtained from three rats treated with eCG before (0 h), 4, and 12 h after hCG injection. Binding activities of EZH2 to the promoter region were analyzed by a ChIP assay. Each bar shows the IP/INPUT ratio. Data are representatives of four independent experiments

To investigate the changes in the recruitment of EZH2 to the promoter regions of *StAR* and *cyp19a1* after ovulation induction, the binding activities of EZH2 to those promoters were examined using a ChIP assay (Fig. 3). The binding activity of EZH2 to the *StAR* promoter region decreased after hCG injection while the binding activity of EZH2 to the *cyp19a1* promoter region increased after hCG injection.

**Fig. 4:**
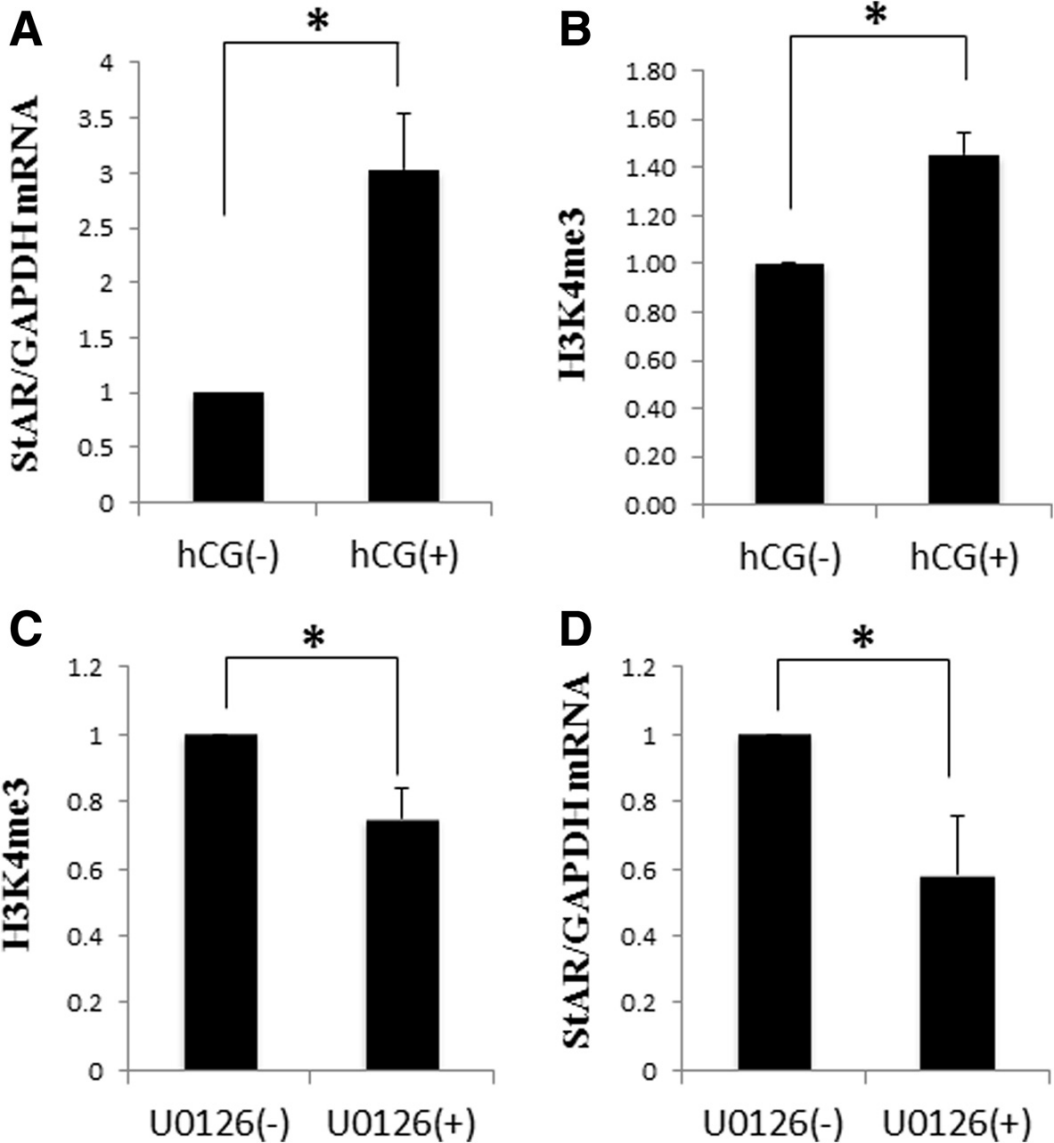
Effects of the inhibitor of ERK-1/2 on H3K4me3 of the StAR promoter region and StAR mRNA expression in granulosa cells undergoing luteinization. Granulosa cells were isolated 48 h after eCG injection, and incubated with or without hCG for 4 h to induce luteinization (**a**, **b**). Granulosa cells were also incubated with or without the inhibitor of ERK (U0126, 10 M) for 2 h prior to hCG stimulation (**c**, **d**). StAR mRNA expression was measured by real-time RT-PCR. The value was normalized to an internal control (GADPH). Data were expressed as a percentage of the group without hCG. H3K4me3 of the StAR promoter region was analyzed by a ChIP assay. The IP/INPUT ratio was calculated and data were expressed as a percentage of the group without U0126. Each bar represents the mean +/− SEM of three independent experiments. *; *p* < 0.05 vs. hCG (−) or U0126 (−)

We also investigated whether ERK-1/2 is involved in the regulation of the histone modification at the *StAR* promoter region and its mRNA expression in granulosa cells undergoing luteinization in vitro. *StAR* mRNA levels were significantly increased by hCG stimulation with the significant increase in H3K4me3 of the *StAR* promoter region (Fig. 4a and b). *StAR* mRNA expression was significantly inhibited by the ERK inhibitor (U0126) accompanied by the significant decrease in H3K4me3 (Fig. 4c and d), suggesting that hCG increases *StAR* gene expression through the ERK-1/2-mediated signaling which is also associated with histone modification (H3K4me3) of the promoter region.

## Discussion

Previous studies have shown that more than 300 genes were up-regulated by more than 4-fold in granulosa cells during ovulation after the ovulatory LH surge [27, 40]. mRNA expression levels of some genes increase by several hundred or even a 1000-fold during this short period. These dramatic changes in gene expression of luteinizing granulosa cells are not only regulated by the activation of several transcription factors, but also by histone modifications [25, 26]. This study showed that the gene expression of histone modification enzymes was altered in granulosa cells undergoing luteinization during ovulation. Among the 84 genes in the PCR array, five (*HDAC4, HDAC10, EZH2, SETDB2*, and *CIITA*) were identified in granulosa cells as histone acetylation- or histone methylation-related genes. In addition, two genes (*NCOA7* and *HP1BP3*) were examined as histone modification-related genes that act downstream of the ERK-1/2 signal. HDAC4, HDAC10, EZH2, SETDB2 and HP1BP3 have transcriptional repression activities, and CIITA and NCOA7 have transcriptional activation activities. All the changes in those histone modification enzymes shown in this study seem to be associated with activating gene expressions. However, it is unclear whether the changes in mRNA expression of the histone modification enzymes are actually related with the promoter specific-histone modification and mRNA expression of the target gene. Further studies are needed to clarify the relationship among the changes in mRNA expression of the histone modification enzymes, the promoter specific-histone modification, and gene expression of the target genes. Although the detailed role and the target genes of the altered histone modification enzymes are unclear, our results suggest that the changes in mRNA expression of the histone modification enzymes play important roles in regulating the expression of various genes during the early stage of luteinization, which may be critical for the subsequent corpus luteum formation.

We recently reported that the H3K27me3 status of the promoter region decreased in the *StAR* gene in granulosa cells undergoing luteinization after ovulation induction while it increased in the *cyp19a1* gene [25]. This change is consistent with the present result that the recruitment of EZH2, which induces H3K27me3, to the promoter region decreased in the *StAR* gene while it increased in the *cyp19a1* gene after ovulation induction. In this study, mRNA levels of *EZH2* decreased in granulosa cells undergoing luteinization after ovulation induction. The changes in mRNA expression of the histone modification enzymes are not always related with the promoter specific-histone modification of the target gene. mRNA levels of the histone modification enzymes are the total amount in the tissues, and do not reflect the local levels of the histone modification in the promoter region of the target gene. The recruitment of the histone modification enzymes to the promoter region is more closely related with the promoter specific-histone modification of the target gene.

Our results showed that ERK-1/2 signal is associated with an increase in H3K4me3, which is an active mark of histone modifications, of the *StAR* promoter region in granulosa cells undergoing luteinization. Although a direct relationship between H3K4me3 of the *StAR* promoter and gene expression was not demonstrated in this study, our results support that hCG increases *StAR* gene expression through the ERK-1/2-mediated histone modification of the promoter region. Regarding how the promoter-specific histone modification is regulated or what kind of histone modification enzymes is preferentially recruited to the promoter region, further studies are needed.

The significant change in mRNA expression of DNA methylation-related genes such as DNMTs (*DNMT1, DNMT3a,* and *DNMT3b*) was not observed among the 84 genes in the PCR array in this study. However, we cannot ignore the possibility that DNA methylation is involved in regulating the expression of various genes in granulosa cells undergoing luteinization after ovulation induction, because the promoter specific-DNA methylation status can be altered even if the gene expression of DNA methylation enzymes did not change [41, 42]. In addition, DNA demethylation including TET should be taken into consideration for understanding the regulation of the DNA methylation [43, 44].

## Conclusions

The present study showed that gene expressions of histone modification enzymes change in granulosa cells undergoing luteinization after ovulation induction. This suggests that histone modifications play important roles in regulating the expression of various genes during the early stage of luteinization, which may be critical for the subsequent corpus luteum formation.

## Abbreviations

CBP: CREB-binding protein
DNMT: DNA methyltransferases
eCG: equine chorionic gonadotropin
ERK: extracellular regulated kinase
hCG: human chorionic gonadotropin
MHC: major histocompatibility complex
